# Molecular Design Using Selected Concentration Effects in Optically Activated Fluorescent Matrices

**DOI:** 10.3390/ijms25094804

**Published:** 2024-04-28

**Authors:** Aneta Lewkowicz, Katarzyna Walczewska-Szewc, Martyna Czarnomska, Emilia Gruszczyńska, Mattia Pierpaoli, Robert Bogdanowicz, Zygmunt Gryczyński

**Affiliations:** 1 Institute of Experimental Physics, Faculty of Mathematics, Physics, and Informatics, University of Gdansk, ul. Wita Stwosza 57, 80-308 Gdańsk, Poland; martyna.czarnomska@phdstud.ug.edu.pl (M.C.); emilia.gruszczynska@phdstud.ug.edu.pl (E.G.); 2 Institute of Physics, Faculty of Physics, Astronomy and Informatics, Nicolaus Copernicus University in Toruń, ul. Grudziądzka 5, 87-100 Toruń, Poland; 3 Department of Metrology and Optoelectronics, Faculty of Electronics, Telecommunication, and Informatics, Gdańsk University of Technology, Gabriela Narutowicza 11/12, 80-233 Gdańsk, Poland; mattia.pierpaoli@pg.edu.pl (M.P.); rbogdan@eti.pg.edu.pl (R.B.); 4 Department of Physics and Astronomy, Texas Christian University, 2995 S. University Dr., Fort Worth, TX 76109, USA; z.gryczynski@tcu.edu

**Keywords:** cyclodimer 1,8-diazafluoren-9-one, friction ridge analysis, absorption spectra, fluorescence spectra

## Abstract

Molecular physics plays a pivotal role in various fields, including medicine, pharmaceuticals, and broader industrial applications. This study aims to enhance the methods for producing specific optically active materials with distinct spectroscopic properties at the molecular level, which are crucial for these sectors, while prioritizing human safety in both production and application. Forensic science, a significant socio-economic field, often employs hazardous substances in analyzing friction ridges on porous surfaces, posing safety concerns. In response, we formulated novel, non-toxic procedures for examining paper evidence, particularly thermal papers. Our laboratory model utilizes a polyvinyl alcohol polymer as a rigid matrix to emulate the thermal paper’s environment, enabling precise control over the spectroscopic characteristics of 1,8-diazafluoro-9-one (DFO). We identified and analyzed the cyclodimer 1,8-diazafluoren-9-one (DAK DFO), which is a non-toxic and biocompatible alternative for revealing forensic marks. The reagents used to preserve fingerprints were optimized for their effectiveness and stability. Using stationary absorption and emission spectroscopy, along with time-resolved emission studies, we verified the spectroscopic attributes of the new structures under deliberate aggregation conditions. Raman spectroscopy and quantum mechanical computations substantiated the cyclodimer’s configuration. The investigation provides robust scientific endorsement for the novel compound and its structural diversity, influenced by the solvatochromic sensitivity of the DFO precursor. Our approach to monitoring aggregation processes signifies a substantial shift in synthetic research paradigms, leveraging simple chemistry to yield an innovative contribution to forensic science methodologies.

## 1. Introduction

The exploration of optically active materials through fluorescence spectroscopy has marked a transformative period in molecular physics, providing a nuanced understanding of molecular interactions and the behavior of complex compounds. Central to this field is the study of molecular structures like 1,8-Diazafluoren-9-one (DFO), whose interactions with α-amino acids present in human eccrine sweat make them instrumental in the visual enhancement of latent fingerprints [1,2]. This specialized application exemplifies a broader scientific theme: the exploitation of specific compound reactivities to detect and analyze molecular residues on various substrates, reflecting the delicate interplay between molecular affinity and fluorescence.

DFO’s interaction with α-amino acids renders it a potent agent for detecting fingerprints on diverse surfaces, outperforming traditional methods in both sensitivity and visibility, especially on porous materials [2,3,4]. The strong affinity of DFO for α-amino acids found in human sweat renders it an effective medium for enhancing the visibility of fingerprints on a variety of surfaces. This technique underscores a significant intersection between physics and chemistry, as it leverages molecular interactions for enhanced visualization and sensitivity in tracing latent prints on porous media, thereby facilitating faster and more sensitive detection at crime scenes [2]. Other methods currently utilized in forensic laboratories, such as IND (1,2-indandione), zinc chloride-enhanced IND (ZnCl2), or ninhydrin, are compared for their efficacy in visualizing dactyloscopic traces and achieving comparable results [2,3,4,5].

Despite the benefits of DFO in latent fingerprint visualization, challenges persist due to the toxicity of methanol, a common solvent for DFO solutions, which poses significant health risks upon inhalation or skin contact [3]. This underscores the necessity for safer latent fingerprint visualization methods that employ less hazardous solvents, thereby minimizing the health risks associated with toxic reagents. Additionally, the analysis of traces on thermal paper, such as receipts and tickets, necessitates specialized methods to preserve the integrity of the substrate. The application of DFO and related compounds like Ninhydrin/Hydrindantin on thermal papers is well documented, with studies aiming to reduce the visual interference caused by these reagents [5,6,7,8]. The incorporation of PVP into the reagent formulation has been shown to diminish the darkening of thermal papers while improving detection sensitivity [6].

In 2019, Lewkowicz’s research group highlighted that replacing methanol with less toxic ethanol in DFO solutions does not adversely affect its spectroscopic properties [9]. Our group has further contributed to this field by demonstrating that DFO molecules can aggregate and thereby reveal dactyloscopic traces on porous substrates such as plain and thermal papers [9,10,11,12]. Our studies from 2019 to 2022 have delved into the fluorescence properties of DFO and its aggregates across different states of matter, uncovering the dynamic responsiveness of DFO aggregation to environmental changes [9,10,11,12].

Our approach, rather than relying on traditional organic synthesis, facilitates product formation through the meticulous control of the original molecule’s environment and the rigidity of its matrix. By regulating parameters such as molecular proximity, steric orientation, and concentration within a tailored environment, we can guide the molecular assembly toward our desired products. This strategy promotes the formation of new molecules, such as via cyclodimers’ formation [13,14,15,16]. This streamlined process reduces the need for superfluous reagents and unwanted byproducts, exemplifying a paradigm shift toward a sustainable and efficient synthetic methodology.

Herein, we demonstrate that the aggregation state of DFO can be selectively tuned in response to its environment, with direct implications for forensic science applications. Our findings reveal that environmental manipulation can foster the formation of the novel cyclodimer 1,8-diazafluoren-9-one (DAK DFO), enhancing friction ridge analysis on porous substrates. In light of the increased interest in the development of superior fingerprint detection reagents, particularly for thermal paper applications, the DAK DFO dimer has exhibited the potential to enhance the sensitivity and contrast of latent fingerprints. Furthermore, the DAK DFO dimer may function as a useful component in security inks for applications in the anti-counterfeiting and product authentication area. In order to enhance security measures, various compounds that are invisible to the human eye can be incorporated into thermal chromic or photochromic security printing inks [17].

This new synthesis pathway highlights our commitment to producing valuable products through eco-friendly methods. DAK DFO has been thoroughly examined using both hands-on experiments and computer-based quantum chemistry, demonstrating the solidity of our research. Altogether, these developments represent a significant step forward in combining molecular spectroscopy with forensic science, paving the way for further research and practical uses.

## 2. Results and Discussion

**Figure 1:**
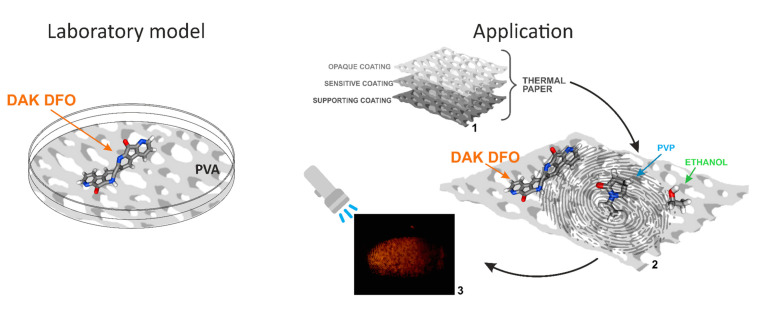
Schematic illustration of two models used in our studies: a laboratory model in which paper is approximated by a PVA matrix, and an application model, where the actual thermal paper (1) is used and the proper environment for “pure chemistry” is provided by applying a DFO–PVP–ethanol solution to the surface (2) and visualisation of dactyloscopic traces (3).

In our previous work, we reported on the tendency of DFO molecules to aggregate [12] and investigated the potential for revealing dactyloscopic traces via such aggregates [11]. Importantly, we demonstrated that the enhancement of fingerprint traces can be achieved through the use of DFO aggregates along with DFO-α-amino acid complexes, which represents a novel approach in the field of forensic science. Our current study builds upon this earlier work and provides further evidence that dactyloscopic traces can be visualized using an effective, non-toxic DFO solution. We also identified the specific form of the aggregate responsible for the observed increase in the luminescence efficiency. The research conducted in this paper demonstrates that the most suitable medium for the formation of the new DAK DFO molecule is a rigid matrix (i.e., paper or a dried PVA polymer film). Accordingly, we present two experimental models: laboratory model rigid media (polymer PVA) and application model rigid media (thermal paper/PVP/ethanol)-Figure 1.

The laboratory model can provide a detailed description of the fluorescence of DAK DFO, including the UV-Vis absorption and fluorescence spectra and intensity decays (fluorescence lifetimes). Using this model, we can better understand the mechanisms that underlie this phenomenon. Furthermore, the specific processes can be verified and elucidated through the use of computational methods. On the other hand, the application model mainly focuses on visual observations of DAK DFO fluorescence.

### 2.1. The Laboratory Model of the Rigid Matrix in the Form of PVA Polymer

Based on our previous findings, which suggest that the aggregation processes of DFO are highly susceptible to external environmental factors [12], we needed a medium that would allow us to precisely control the experimental conditions—especially the rigidity of the medium and the DFO concentration. We selected a matrix of polymerized polyvinyl alcohol (PVA) as an ideal medium because it contains -OH groups in the monomer that help disperse DFO inside the polymer network. Moreover, changing the concentration of PVA affects the rigidity of the medium.

**Figure 2:**
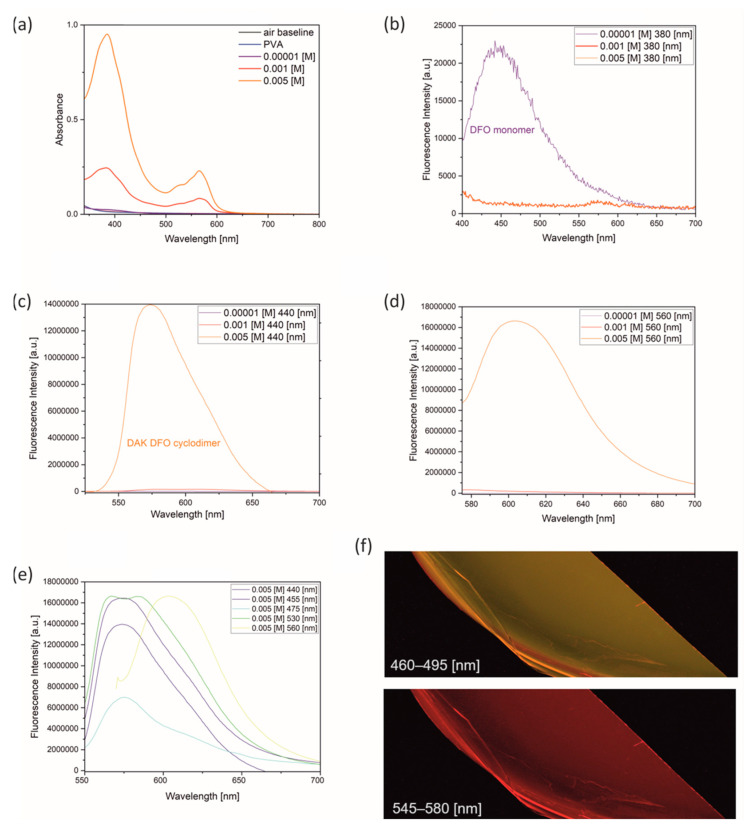
Absorption and fluorescence spectra of DFO in PVA polymer. Absorption spectra at concentrations 1 × 10^−5^, 1 × 10^−3^, and 5 × 10^−3^ M (**a**). Fluorescence spectra at varying dye concentrations for excitation wavelengths of 380 nm (**b**), 440 nm (**c**), and 560 nm (**d**). Fluorescence spectra at 5 × 10^−3^ M for different excitation wavelengths (**e**). Stereoscopic image of fluorescence at 5 × 10^−3^ M concentration (a scale of 1:1), illustrating the response of DFO in the PVA polymer to different excitation wavelengths (**f**).

The absorption and fluorescence spectra were measured to provide evidence of the formation of a new DAK DFO structure in the PVA matrix. The absorption spectra obtained for different concentrations of DFO in PVA are presented in Figure 2a. The most significant changes were observed at concentrations of 1 × 10^−3^ [M] and 5 × 1 × 10^−3^ [M], where an additional absorption band with well-defined maxima around 520 nm and 570 nm was detected, suggesting the presence of structural forms other than DFO, which exhibited maximum absorption at around 380 nm. In Figure 2b–e, the dependence of the emission of the DFO/PVA films obtained on the excitation wavelength is shown. Surprisingly, all samples with a concentration of 5 × 10^−3^ [M] exhibited fluorescence at a maximum of around 580 nm, despite using only the DFO molecule to obtain them, which should theoretically emit at around 450 nm. These results clearly suggest the formation of new structural forms that are stable despite the changes in the excitation wavelength. The new forms exhibit different emission properties from those of the original molecule.

**Figure 3:**
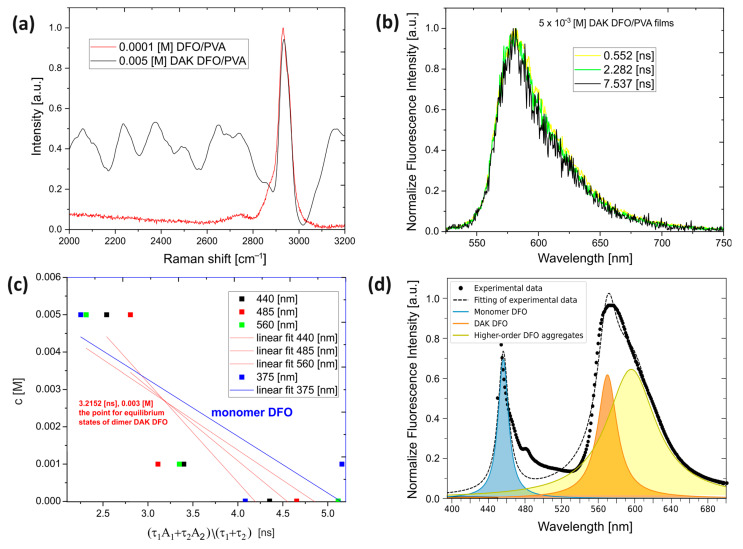
Raman spectra for DFO monomer and DAK DFO dimer (**a**). Time-resolved emission spectra for DAK DFO, normalized fluorescence intensity for 0.5552 [ns], 2.282 [ns], and 7.537 [ns] for excitation wavelength 530 nm (**b**). Fluorescence lifetime for various excitation wavelengths and low and high dye concentrations, alongside the observed point indicating the equilibrium state between dimer DAK DFO and other aggregates that form DFO during time-resolved emission spectra (exp = 2) (**c**). Fluorescence spectrum deconvolution. The graph shows normalized fluorescence intensity versus wavelength, distinguishing contributions from the DFO monomer, DAK DFO cyclodimer, and higher-order DFO aggregates (**d**).

Figure 2c shows a noteworthy observation that at higher concentrations, a new fluorescence band emerged for excitation wavelengths of 440 nm and above. This observation indicates the formation of a structured form that remains stable at an excitation wavelength of 440 nm or higher. Moreover, the time-resolved fluorescence spectra for the initial DAK DFO concentration of 5 × 10^−3^ [M] (Figure 3b) fully illustrate the stability of the new chemical formulation obtained. The emission maximum did not shift with time, and no new short-lived forms appeared. This suggests the existence of a single (one type) emitting center, rather than an aggregate of several centers.

Spectroscopic analysis of the rigid matrix model described above indicates that we can create a new chemical structure, an aggregate, through “pure chemistry” by adjusting the concentration of DFO and the rigidity of the matrix. Since the newly formed molecule was very stable over time, both in PVA and in the structure of thermal paper, we postulate that two DFO molecules were joined in the cycloaddition reaction pathway, which is well known for this type of chemical structure [13,14,18] (more details are provided in Section 2.3). However, our approach to constructing the synthesis pathway and fulfilling the technical requirements of the reaction is entirely novel.

Figure 2 confirms that DAK DFO is an excited cyclodimer, which is formed only when the dimer components are in rigid media at a high concentration of 5 × 10^−3^ [M]. Therefore, its existence can only be proven based on fluorescence measurements. The fluorescence wavelength of the excited cyclodimer is longer (lower energy) than the emission of the excited monomer. Furthermore, the formation of the excited cyclodimer DAK DFO is dependent on molecular interactions. In our experiment, we found that the optimal concentration for DAK DFO formation occurred at 5 × 10^−3^ [M] due to the high density of the monomer. At an excitation wavelength of 455 nm, DAK DFO appeared to emit an orange light, but at an excitation wavelength of 560 nm, higher aggregates of DFO appeared to emit an orange light (Figure 2f).

To further confirm the presence of the cyclodimer DAK DFO in a rigid matrix, we investigated the fluorescence properties of DFO under different excitation conditions and concentrations. Our results showed that the optimal excitation wavelength for the highest fluorescence intensity was 440–530 nm, with the maximum fluorescence band occurring at 580 nm (Figure 2e). In contrast, a significant fluorescence intensity was observed at an excitation wavelength of 560 nm, with the maximum fluorescence band occurring at 620 nm for the same dye concentration 5 × 10^−3^ [M], which was attributed to the formation of higher-order aggregates. At low concentrations, we observed the fluorescence lifetime characteristic of the DFO monomer, whereas the fluorescence lifetimes corresponding to DAK DFO were observed at the highest concentration. The fluorescence intensity decay was observed for each excitation wavelength in at least two forms. For instance, upon 380 nm excitation, we observed fluorescence intensity decay for both the DFO monomer and the forms formed by interaction with the OH groups present in the PVA polymer. The solvatochromic susceptibility of DFO was confirmed earlier. In contrast, upon excitation at 455 nm and 560 nm, we observed fluorescence intensity decays from different forms compared to 380 nm excitation; along with the monomer form of DFO, we observed the formation of DAK DFO. We chose 455 nm as the excitation limit based on the results of the DAK DFO fluorescence observation in our application model—thermal paper.

Raman spectroscopy investigations have affirmed the structure of the dimer proposed through spectrophotometric and spectrofluorimetric measurements in both steady-state and time-resolved modes, as well as quantum mechanical calculations. The study presents results in the form of Raman scattering ranging from 2000 cm^−1^ to 3200 cm^−1^ (see Figure 3a), revealing a distinctive “fingerprint” for the DAK DFO dimer molecule. Particularly for DAK DFO, we observed a robust capability for fluorescence within the VIS range, thereby measurements from 0 to 2000 cm^−1^ were significantly affected by this phenomenon, and interpretation within this range was notably disrupted by the fluorescence background [19].

Conversely, in the range of 2000 cm^−1^ to 3200 cm^−1^, the majority of the observed Raman scattering lines pertain to the chromophore of the dimer [20]. In this region, the spectra of the monomer and DAK DFO in their ground states diverge significantly. For the DAK DFO dimer, characteristic doublet lines emerged, indicating the presence of various C-H bond conformations within the cyclobutane plane that form the connection between the monomer structures, ultimately yielding the DAK DFO dimer [19,20]. Raman spectra, particularly focusing on the C-H, N-H, and O-H stretching regions (2700–3600 cm^−1^), provide conclusive evidence of the cyclodimer structure. This analysis confirms the presence of C-H groups in the cyclobutane group, a structure that forms during the cycloaddition of DFO molecules [21,22,23,24]. Nevertheless, since our experimental PVA matrices incorporate water into their structure, FTIR experimental measurements are heavily distorted and the interpretation of the study results is subject to substantial error. Thus, the quantum mechanical simulations of IR spectra, which are not subject to this error, are presented in this work (see Supplementary Materials, Table S2).

**Figure 4:**
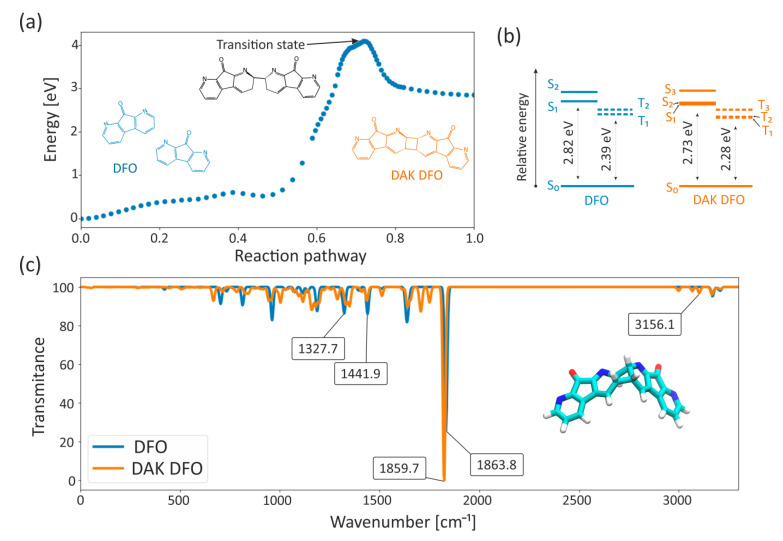
The Nudged Elastic Band reaction pathway of DFO cycloaddition (**a**). IR spectra of DFO (blue) and DAK DFO (orange) in ethanol solution (implicit solvent model). The spectra represent computed intensities rescaled to 100 plotted against frequencies, with a Gaussian broadening (fwhm = 15 cm^−1^) (**b**). Jablonski diagram showing the energy levels of singlet and triplet states for DFO and DAK DFO in ethanol (**c**). It is important to keep in mind that these energies are the energy of the state in the ground state geometry.

The overarching molecular theory concerning the complementarity of IR absorption spectra and Raman scattering also validated the presence of C-H vibrations of varying conformations in the plane in the previously postulated “fingerprint” region of the DAK DFO dimer. These C-H vibrations in the cyclobutane belonging to the DAK DFO structure and their absence in the monomer structure are outlined (see Table S2 and the IR spectrum in Figure 4b).

Moreover, the stable structure of the dimer was also corroborated by time-resolved fluorescence spectra, where, despite changes in the excitation wavelength ranging from 450 nm to 520 nm, a consistent fluorescence spectrum was obtained. Furthermore, the time-resolved spectral evolution demonstrated a uniform fluorescence structure, as both the optical contour of the band and the maximum fluorescence band remained unaltered over time, as illustrated in Figure 3b.

### 2.2. The Theory of Equilibrium States in Time-Resolved Spectra

As the excitation wavelength increased, we observed alterations in average lifetimes along with concentration changes. These alterations, however, exhibit a linear dependency. Moreover, the determined average fluorescence lifetimes (Table S1), obtained through an exp = 2 fitting, affirm the presence of two forms, namely the DFO monomer and the DAK DFO dimer. We illustrated these dependencies in Figure 3c, where the formation of a point was observed for the linear fitting of the dependence:

$$f\left(\frac{t_{1}A_{1}+t_{2}A_{2}}{t_{1}+t_{2}}\right)=c$$

It was found that if the set of f(t) = c relationships intersect at a single point, representing a true equilibrium, the point can be identified by analyzing the fluorescence intensity decays for different excitation wavelengths of 440 nm, 485 nm, and 560 nm observed at the same emission band maximum of 580 nm (postulated for the DAK DFO dimer). There is reasonable evidence that emitting species are present in the studied system, including DFO monomer (after excitation wavelength 380 nm), DAK DFO dimer (after excitation wavelength 440–485 nm and still present after excitation wavelength 530 nm), and higher aggregates (after excitation wavelength 560 nm), and that the determined point represents the optimal concentration of the first DAK DFO dimer in the rigid material under study.

In the case of molecules forming within rigid matrices and possessing short lifetimes, only the time-resolved spectra and decay of fluorescence band intensity can confirm their presence, creating dependencies as depicted in Figure 3c. Stationary spectra, burdened with prolonged measurement times, preclude the separation and presentation of equilibrium states present in rigid materials. While stationary spectra provide our initial premise for the formation of aggregate forms, our conclusive argument is gleaned from the time-resolved spectra.

### 2.3. Explanation of the Underlying Chemistry Using Computational Chemistry Methods

Based on previous studies on the DFO molecule, we have gained extensive knowledge of its chemical behavior in various environments, including its photophysical [9,10,11,12] and application properties [11]. Our investigations revealed clear distinctions between the DFO monomer molecule, its complex with α-amino acids, and the aggregate formed as the postulated cyclodimer. In a rigid matrix, the emission spectrum of DAK DFO at 455 nm excitation (with the highest fluorescence intensity) has a fluorescence maximum of 580 nm. The position of the band maximum for DAK DFO’s fluorescence is similar to that of the DFO complex with glycine. However, the optimal excitation wavelength for the DFO-glycine complex is 530 nm, indicating that the emission does not originate from the complex but from the newly formed molecule. Based on the observed stability of the new molecule over time in both PVA and the structure of thermal paper, we propose that two DFO molecules underwent a cycloaddition reaction pathway to form the new DAK DFO molecule.

To identify the reaction mechanism and possible transition states, we performed quantum chemical calculations using the Nudged Elastic Band [25] (Figure 4a). In typical reaction pathways, a cycloaddition reaction to the C=C bond is expected. However, in the case of a DFO molecule that contains an N=C double-bond mode with N embedded in the ring, a preference for cycloaddition to C involved in the N=C bond was observed, leading to the formation of a cyclodimer. Standard organic syntheses conducted under controlled laboratory conditions have already demonstrated the possibility of various cycloaddition reactions occurring through the excited states of molecules [15,16]. In our research, we observed the formation of the proposed DAK DFO compound in favor of the ortho adduct over the meta adduct, following the region-selectivity rule. This is due to an increase in the amount of charge transfer occurring in the excited DAK DFO cyclo-dimer. The transition form is formed by regrouping electrons around the nitrogen atom, resembling the ionic-type reaction of molecules in the ground state. The final product is formed as a consequence of the circular displacement of pi electrons. Two unsaturated DFO molecules underwent dimerization at positions 7 and 8 in 1,8-diazafluoren-9-one (see Supplementary Materials—Video S1 and Figure S1). The DFO molecule changes the electronic distribution around the positional skeleton of the molecule, resulting in the observation of DAK DFO dimers at high concentrations.

In Figure 4c, we presented numerically calculated Jablonski diagrams for two chemical compounds, DFO and DAK DFO, in a polar environment of ethanol. Based on these diagrams, we can see that the energy donors are in the triplet state, which can sensitize the cycloaddition reaction. Therefore, we hypothesize that the reaction most likely occurs in the triplet state of the molecule. It is worth noting that the substances that quench the triplet states do not reduce the quantum yield of fluorescence, suggesting that regrouping to the cyclodimer must be a very fast process. Our analysis revealed that DAK DFO exhibits greater photochemical competitiveness than DFO. The difference between the ground state and the first excited state is smaller for DAK DFO, meaning that in an environment favorable for the formation of DAK DFO, these molecules will absorb light radiation more easily compared to DFO. In other words, less energy is required to excite DAK DFO, as the light of a shorter wavelength is sufficient to induce excitation. Notably, experimental investigations conducted on authentic thermal papers revealed that the most optimal visualization of dactyloscopic traces was achieved utilizing an excitation wavelength of 455 nm (see Section 2). This particular wavelength corresponds to the theoretically determined energy difference between the ground and excited states in DAK DFO, which has been estimated to be 2.73 eV.

To confirm the structure of DAK DFO, quantum chemical calculations with B3LYP hybrid density functional [26] were carried out for optimization and to model its vibrational spectra (see Figure 4b). The figure shows that the analyzed molecules exhibit similar optical properties in the oscillatory–rotational absorption range. Nevertheless, some important differences can be noticed. The N-H bond is less polar than the OH bond, which leads to a weaker absorption band for N-H, with a narrower width and a position at 3047.7 cm^−1^, 3093.4 cm^−1^, and 3000 cm^−1^ for DAK DFO. Furthermore, the C=O stretching of the ketone group is observed at approximately 1863.8 cm^−1^ for DFO and 1859.7 cm^−1^ for DAK DFO. Other double bonds, such as C=C and C=N, exhibit absorbance in the lower frequency range of approximately 1550–1650 cm^−1^. The C=C stretching of the benzene ring is indicated by two sharp absorption bands, one at ~1441.9 cm^−1^ and one at 1327.71 cm^−1^ for DFO, and 1438.8 cm^−1^, 1328.9 cm^−1^, and 1280 cm^−1^ for DAK DFO.

It is important to note that the IR spectrum region with lower frequencies between 400 and 1400 cm^−1^ is known as the fingerprint region. Similar to a human fingerprint, the pattern of absorbance bands in this region is a unique characteristic of the compound as a whole. If two different molecules have the same functional groups, their IR spectra will still differ and this difference will be reflected in the bands in the fingerprint region. This allows for IR spectra of unknown samples to be compared to a database of IR spectra of known standards, providing a means of confirming the identification of the unknown sample.

Our experimental and computational analyses have converged to the conclusion that we have discovered a novel DFO dimer resulting from cycloaddition to the C=N bond in the aromatic ring. Notably, the cyclodimer or its potential formation has never been reported before in the context of revealing dactyloscopy traces on paper using a solution with DFO. Our work presents the first evidence of its existence. Previous challenges in interpretation arose from the striking structural similarity between the DFO complex with glycine and the DAK DFO cyclodimer, including the presence of four nitrogens in the aromatic ring and the same number of aromatic rings with a comparable electron configuration, specifically the chromophore groups. As a result, we observed a similar excitation energy value for the DFO complex with glycine and the DAK DFO dimer. This argument provides strong evidence for comparable spectroscopic properties, particularly the fluorescence characteristics of these two species. Our findings offer valuable insights into the chemical behavior of DFO and expand our understanding of its potential applications.

### 2.4. Practical Application in Friction Ridge Analysis

The findings from our laboratory model and theoretical calculations have helped to elucidate the phenomena that occur when using DFO in practical applications. We have observed that in a rigid matrix, a stable cyclodimeric form is generated at a concentration of 0.001 [M] DFO/PVA, with an absorption and emission spectrum that closely resembles the DFO complex with α-amino acids. This novel form of DFO has also been detected when revealing fingerprints on paper and thermal paper. These results can be attributed to the naturally rigid and porous nature of paper, where a proper concentration of DFO and the use of PVP as a stabilizing polymer provide suitable conditions for the formation of DAK DFO.

Practical observations made during the visualization of fingerprints on thermal paper, as illustrated in the schematic and the fingerprint in Figure 1, align with the results shown in Figure 2b for the laboratory model. The optimal visualization effect, marked by the highest fluorescence intensity from the fingerprint surface, was obtained using a forensic illuminator with an excitation wavelength of 455 nm. Although there were differences in the model composition, the overall conditions for the formation of the new aggregate form were similar.

DAK DFO is a highly effective method for revealing fingerprints on thermal paper, owing to its spectroscopic properties as well as its chemical structure. Thermal paper is known for its complex structure (refer to Figure 1). Modern thermal papers are typically based on leuco-dye chemistry, as described in detail by Kim et al. (2018) [27]. When a developer (usually Bisphenol A or Bisphenol S) reacts with the fluoran ring, it forms a visible black dye. DAK DFO features an amine group that has both alkaline properties and polarity. This compound is designed to prevent ring banding in thermal dyes due to its alkaline properties while also facilitating a smooth paper coating structure because of its greater polarity than DFO. The addition of an amino group to the ring structure significantly stabilizes the molecule during interactions with α-amino acids or leuco dyes, where it serves as their deactivator.

## 3. Methods and Materials

### 3.1. Chemicals and Materials

The chemicals used in this study were 1,8-diazafluoren-9-one (DFO) obtained from Sigma-Aldrich (Munich, Germany) and ethanol obtained from POCH Company (Gliwice, Poland). Kollidon 12 PF-polyvinylpyrrolidone (PVP) was obtained from the BASF Chemical Company (Ludwigshafen, Germany), and polyvinyl alcohol (PVA) was obtained from Sigma Aldrich (Munich, Germany).

### 3.2. Preparation

DFO/PVA films were prepared by dissolving DFO and PVA (4%) in demineralized water at different concentrations of DFO (1 × 10^−4^, 1 × 10^−3^, 5 × 10^−3^, and 1 × 10^−2^ [mol/dm^3^]) at room temperature and atmospheric pressure.

The working solution for visualizing fingerprint traces on thermal papers was prepared using DFO, PVP polymer, and ethanol. Further details can be found in the patent description [P.443382—the number of the National Patent notice].

### 3.3. Characterization Techniques

The absorption spectra of the DFO/PVA films were recorded using a Shimadzu UV-1900i UV-VIS spectrophotometer (Shimadzu, Kyoto, Japan). Fluorescence spectra were measured using a spectrofluorometer FluoroMax-4P TCSPC (Horiba, Osaka, Japan) with a 150 W Xenon lamp emitting in the 220–850 nm range. Time-resolved emission spectra (TRES) were recorded using a pulsed spectrofluorometer (2501S Spectrograph, Bruker, Optics Inc., Billerica, MA, USA), as described in [25]. The fluorescence intensity decays and lifetime measurements were performed using a FluoTime 300 fluorescence lifetime spectrometer (PicoQuant, GmbH, Berlin, Germany).

The emission of samples in the friction ridge analysis was obtained using a forensic illuminator with an excitation wavelength of 455 nm [Labino Nova Torch—bandwidth 440–460 nm, typical output 23 lm, 354 mW] and Olympus SZX16 stereo microscope (Olympus, Tokyo, Japan) with fluorescence and digital camera with CellSens Standard software (CS-ST-V3 Standard V3).

The Raman spectra were measured using the Raman spectrometer (LabRam Aramis Raman spectrometer from Horiba Jobin Yvon, equipped with an Olympus BX41 confocal microscope, and a Synapse CCD camera from Horiba Jobin Yvon, Tokyo, Japan). Excitation was performed with a 632 nm wavelength of a Melles Griot helium–neon laser; the spectral resolution was equal to 2 cm^−1^ in the range of 2000–3200 cm^−1^ with an integration time of 5 s (20 averages); the diffraction grating had 300 lines mm^−2^.

### 3.4. Computational Methods

All calculations were performed using the ORCA 5.0.3 [28,29] software package. All structures were first optimized using the B3LYP functional [26] and the def2-SVP basis set [30]. The RIJCOSX approximation was used for Coulomb integrals and numerical integration for HF exchange [31]. To include solvent effects in our calculation, we used a conductor-like polarizable continuum model [32] with a refractive index and dielectric constants of 1.361 and 24.3 for ethanol. For the excited states, TDDFT was employed [33]. We searched for the first 10 roots in both the singlet and triplet states. The convergence criteria for both the SCF and geometry optimizations were set to TIGHT, and all the other parameters were chosen as the default. The theoretical prediction of the reaction pathway was made using the Nudged Elastic Band (NEB-TS) method [25]—a widely used computational approach to identifying the reaction pathway between two known states of a molecular system. NEB constructs a series of intermediate states between the initial and final states and relaxes the atomic coordinates at each intermediate state to find the minimum energy pathway.

The deconvolution analysis in Figure 3d was performed by fitting a combination of three Gaussian functions to the spectral data using the scipy.optimize.curve_fit method from the SciPy library in Python 3.7 [34]. Each Gaussian function was defined with variable parameters for amplitude, center, and width to account for the respective shape and size of the spectral features attributed to the individual species. The optimization process iteratively adjusted these parameters to minimize the difference between the experimental data and the fitted curve. The data were normalized to facilitate comparison between the components, and the deconvolution results were visualized by plotting both the raw data and the fitted Gaussians, allowing for a clear representation of the relative contributions of each molecular form to the total emission spectrum.

### 3.5. Research Permission and Ethics Declarations

This study was approved by the Ethics Committee of the University of Gdańsk. Full informed and written consent was obtained from the participants before the initiation of the study for study participation and for publication of the pictures used in the manuscript. The experimental protocol was approved by the University of Gdańsk, and all methods were performed according to the relevant guidelines and regulations.

## 4. Conclusions

The ability to synthesize highly complex molecules has been accompanied by the production of numerous secondary products, which can reduce the efficiency of the primary reaction due to competing parallel reactions. Our approach is a novel synthesis method that emphasizes high chemical yields while minimizing the use of additional substrates and toxic byproducts. The method achieves product formation through controlled aggregation processes and offers several advantages, including its non-toxicity and safety for use, as well as its simplicity and lack of requirement for sophisticated equipment. Thus, the method has the potential to be widely used in various fields and is not limited to forensic sciences.

Experimental and computational analyses reveal that the cycloaddition of two DFO molecules leads to the formation of a novel DAK DFO dimer. This reaction involves the formation of two bonds—one between the carbon atoms of the C=N bond, and one between the C=C atoms of the aromatic rings. It is worth noting that the formation of the cyclodimer, or its potential formation, has never been reported in previous studies investigating dactyloscopy traces on paper using a DFO solution. Therefore, our work provides the first evidence of its existence. We characterized the spectroscopic properties of the newly discovered compound in the model rigid medium in the form of the PVA film and showed that DAK DFO can also be identified during the visualization of fingerprints on both regular paper and thermal paper.

It is worth mentioning that the experimental investigations carried out on genuine thermal papers have demonstrated that the most effective visualization of dactyloscopic traces occurred when using an excitation wavelength of 455 nm. This particular wavelength aligns with the theoretically calculated energy difference between the ground and excited states in DAK DFO, which has been estimated to be 2.73 eV.

The new procedure described in this study enables the visualization of prints in a previously unexplored manner by aggregating DFO particles in the reaction environment. This represents a significant contribution to the field of forensic science and opens up new analytical possibilities. Previous attempts to identify friction ridges on thermal paper were successful, but they utilized toxic and invasive solutions, particularly at higher staging on thermal paper, as noted in a study by Luo et al. [6]. Our approach introduces a completely new compound, DAK DFO. The new compound features an amine group that displays alkaline properties and polarity. This chemical effectively stops ring banding in thermal dyes due to its alkaline nature while also improving the paper coating structure due to its higher polarity compared to DFO. Additionally, the inclusion of an amino group in the ring structure of DAK DFO significantly boosts its stability during interactions with α-amino acids and leuco dyes, where it serves as a deactivator.

Developing an appreciation of the implications of using a particular representation of molecule concentrations—including the existence of the ability to obtain new structural forms while controlling the spectroscopic properties of the optically modeled active materials—is the key to their successful implementation. We demonstrate a completely new approach to the possibility of controlling the aggregation processes to obtain the relevant spectroscopic properties of the materials.

The current limitations of obtaining optically active materials in this way are due to a completely new concept of synthesis. The molecular design aimed to follow the model of organic synthesis, often requiring difficult reaction conditions, high temperatures, complex laboratory equipment, and toxic reaction environments.

The proposed procedure using spectroscopic control of aggregation phenomena to obtain structures with the required spectroscopic properties has a basic limitation, i.e., the correct knowledge of molecular physics and scientific correctness when performing measurements on stationary and time-resolved molecular spectroscopy apparatus.

We hope that our work will inspire further research and development of innovative and non-toxic chemical methods for advancing scientific fields and improving human well-being.

## Data Availability

All input files for quantum chemical computations and optimized structures of reactants, the transition state, and the DAK DFO molecule can be found in our GitHub repository: https://github.com/kszewc/DAK. All further data, including experimental results, are deposited as the open data set at Lewkowicz, A. (2023) (absorption and fluorescence spectra of 1,8-diazafluoren-9-one (DFO) in PVA polymer for different concentrations [Data set]). Gdańsk University of Technology. https://doi.org/10.34808/hzze-mj81.

## Supplementary Materials

The following supporting information can be downloaded at: https://www.mdpi.com/article/10.3390/ijms25094804/s1.
