# Supplementary material for: Molecular Design Using Selected Concentration Effects in Optically Activated Fluorescent Matrices
Source: Int J Mol Sci. 2024 Apr 28;25(9):4804. doi: 10.3390/ijms25094804 (PMC11084466; doi:10.3390/ijms25094804)
Supplement: Supplementary file 1 [file ijms-25-04804-s001.zip › SI_DAK_DFO_Lewkowicz IJMS.pdf]

SUPPLEMENTARY INFORMATION:

" Molecular design using selected concentration effects in optically activated fluorescent matrices ".

Aneta Lewkowicz[1*], Katarzyna Walczewska-Szewc[2*], Martyna Czarnomska[1], Emilia Gruszczyńska[1], Mattia Pierpaoli[3], Robert Bogdanowicz[3], Zygmunt Gryczyński[4]

1. Faculty of Mathematics, Physics, and Informatics of the University of Gdansk, Institute of Experimental Physics, ul.Wita Stwosza 57, 80-308 Gdańsk, Poland; *email: aneta.lewkowicz@ug.edu.pl,
2. Institute of Physics, Faculty of Physics, Astronomy and Informatics, Nicolaus Copernicus University in Toruń, ul. Grudziądzka 5, 87-100 Toruń, Poland; *email: kszewc@umk.pl
3. Department of Metrology and Optoelectronics, Faculty of Electronics, Telecommunication, and Informatics, Gdańsk University of Technology, Gabriela Narutowicza 11/12, Gdańsk 80-233, Poland
4. Department of Physics and Astronomy, Texas Christian University, 2800 S. University Dr. Fort Worth, Texas, 76129, USA.

Table S1. The mean fluorescence lifetime (amplitude weighted, $\tau_{medium}=\left(\frac{t_1A_1+t_2A_2}{t_1+t_2}\right)$) of DFO in PVA films. λ_{obs} = 580 nm; λ_{ex} = 380 nm, 455 nm, and 560 nm.

**c [M]**	**τ_{medium} [ns] 380 [nm]**	**x^2**	**τ_{medium} [ns] 455 [nm]**	**x^2**	**τ_{medium} [ns] 560 [nm]**	**x^2**
0.0001	4.082	1.04	4.352	0.973	5.115	0.912
0.001	5.157	1.12	3.398	0.962	3.349	0.955
0.005	2.252	1.03	2.540	0.918	2.312	0.930

Table S2. Detailed IR spectra information of DFO and DAK DFO in ethanol solution (implicit solvent model).

DFO in ethanol				DAK DFO in ethanol			
Mode	Freq [cm -1]	Eps [L/(mol*cm)]	Int [km/mol]	Mode	Freq [cm -1]	Eps [L/(mol*cm)]	Int [km/mol]
6	99.50	0.000015	0.08	6	23.13	0.000053	0.27
7	128.16	0.000000	0.00	7	36.54	0.000601	3.04
8	149.19	0.000043	0.22	8	54.78	0.001139	5.76
9	222.22	0.000028	0.14	9	78.21	0.000022	0.11
10	287.74	0.000000	0.00	10	104.59	0.000034	0.17
11	292.34	0.000620	3.13	11	132.01	0.000225	1.14
12	421.24	0.001308	6.61	12	141.16	0.000070	0.36
13	423.75	0.000491	2.48	13	153.09	0.000285	1.44
14	444.30	0.000023	0.12	14	171.48	0.000101	0.51
15	457.44	0.000000	0.00	15	204.65	0.000019	0.10
16	510.43	0.000050	0.25	16	214.40	0.000077	0.39
17	571.28	0.000219	1.11	17	236.71	0.000144	0.73
18	587.42	0.000000	0.00	18	262.43	0.000173	0.87
19	634.42	0.000255	1.29	19	286.64	0.001149	5.81
20	701.84	0.002682	13.55	20	309.77	0.000372	1.88
21	703.61	0.009091	45.94	21	319.80	0.000437	2.21
22	733.09	0.003052	15.42	22	339.80	0.000484	2.45
23	792.30	0.000000	0.00	23	386.82	0.000356	1.80
24	813.09	0.012149	61.40	24	406.62	0.000143	0.72
25	819.10	0.000750	3.79	25	428.92	0.000216	1.09
26	859.35	0.000000	0.00	26	440.02	0.000340	1.72
27	886.66	0.000743	3.76	27	448.08	0.001039	5.25
28	961.19	0.022867	115.56	28	481.12	0.000077	0.39
29	971.24	0.000207	1.05	29	503.88	0.001894	9.57
30	976.92	0.000000	0.00	30	514.28	0.001225	6.19
31	1026.89	0.000185	0.94	31	525.52	0.000494	2.50
32	1027.29	0.000000	0.00	32	543.67	0.000555	2.80
33	1028.84	0.001797	9.08	33	561.10	0.000186	0.94
34	1064.47	0.000003	0.02	34	573.31	0.000443	2.24
35	1074.95	0.003641	18.40	35	586.69	0.000236	1.19
36	1113.33	0.004464	22.56	36	601.28	0.000779	3.94
37	1139.95	0.003551	17.95	37	622.26	0.000516	2.61
38	1170.90	0.000185	0.93	38	648.43	0.000406	2.05
39	1187.09	0.016707	84.43	39	664.64	0.007191	36.34
40	1245.90	0.000518	2.62	40	669.61	0.008411	42.51
41	1289.67	0.000015	0.08	41	692.68	0.000672	3.39
42	1312.38	0.004214	21.30	42	711.53	0.004029	20.36
43	1323.86	0.017336	87.61	43	718.18	0.001562	7.89
44	1395.92	0.002552	12.90	44	757.03	0.000526	2.66
45	1437.15	0.000130	0.66	45	770.70	0.001632	8.25

46	1439.83	0.018483	93.41	46	782.12	0.002751	13.90
47	1501.12	0.000034	0.17	47	786.50	0.002764	13.97
48	1509.40	0.001267	6.41	48	823.12	0.004308	21.77
49	1633.30	0.012763	64.50	49	830.00	0.003610	18.24
50	1638.60	0.001203	6.08	50	842.64	0.006290	31.79
51	1640.52	0.013749	69.48	51	854.94	0.001081	5.46
52	1649.06	0.003115	15.74	52	874.48	0.001129	5.71
53	1832.99	0.099222	501.43	53	877.69	0.000542	2.74
54	3169.35	0.006400	32.34	54	901.92	0.002014	10.18
55	3169.46	0.000021	0.10	55	917.54	0.002382	12.04
56	3194.65	0.000880	4.44	56	943.88	0.013897	70.23
57	3196.54	0.000152	0.77	57	958.46	0.011767	59.47
58	3208.29	0.002087	10.55	58	976.95	0.000171	0.87
59	3208.98	0.000401	2.03	59	978.32	0.000097	0.49
				60	986.90	0.003757	18.99
				61	1003.33	0.016333	82.54
				62	1025.63	0.002440	12.33
				63	1031.96	0.000087	0.44
				64	1032.77	0.000045	0.23
				65	1041.15	0.001894	9.57
				66	1065.70	0.000875	4.42
				67	1069.72	0.004357	22.02
				68	1087.09	0.003424	17.30
				69	1096.25	0.008212	41.50
				70	1115.00	0.014280	72.17
				71	1123.46	0.002680	13.54
				72	1134.60	0.002838	14.34
				73	1155.77	0.014865	75.12
				74	1162.65	0.008086	40.86
				75	1166.30	0.007588	38.35
				76	1178.24	0.007435	37.57
				77	1182.04	0.012882	65.10
				78	1195.67	0.006541	33.05
				79	1198.16	0.009871	49.89
				80	1221.87	0.003272	16.54
				81	1255.53	0.002739	13.84
				82	1284.07	0.006611	33.41
				83	1291.40	0.008512	43.01
				84	1297.79	0.000081	0.41
				85	1308.36	0.000604	3.05
				86	1323.50	0.004312	21.79
				87	1329.65	0.009540	48.21
				88	1334.74	0.007856	39.70
				89	1346.50	0.005408	27.33
				90	1349.70	0.011332	57.27
				91	1353.07	0.004037	20.40
				92	1384.81	0.000420	2.12
				93	1410.24	0.004998	25.26
				94	1434.68	0.006466	32.68
				95	1437.76	0.008124	41.05
				96	1511.40	0.004952	25.03
				97	1512.63	0.004500	22.74
				98	1613.67	0.000967	4.89

				99	1615.95	0.001915	9.68
				100	1639.25	0.011855	59.91
				101	1640.69	0.008424	42.57
				102	1655.52	0.012757	64.47
				103	1702.45	0.009763	49.34
				104	1708.71	0.018919	95.61
				105	1750.54	0.016363	82.69
				106	1821.76	0.076299	385.58
				107	1824.67	0.133375	674.02
				108	2984.82	0.000353	1.79
				109	2999.60	0.004080	20.62
				110	3067.91	0.004364	22.05
				111	3102.72	0.006542	33.06
				112	3164.83	0.003649	18.44
				113	3166.02	0.003673	18.56
				114	3170.45	0.000952	4.81
				115	3182.44	0.001405	7.10
				116	3195.01	0.000323	1.63
				117	3195.06	0.000319	1.61
				118	3209.16	0.001206	6.10
				119	3209.24	0.001104	5.58

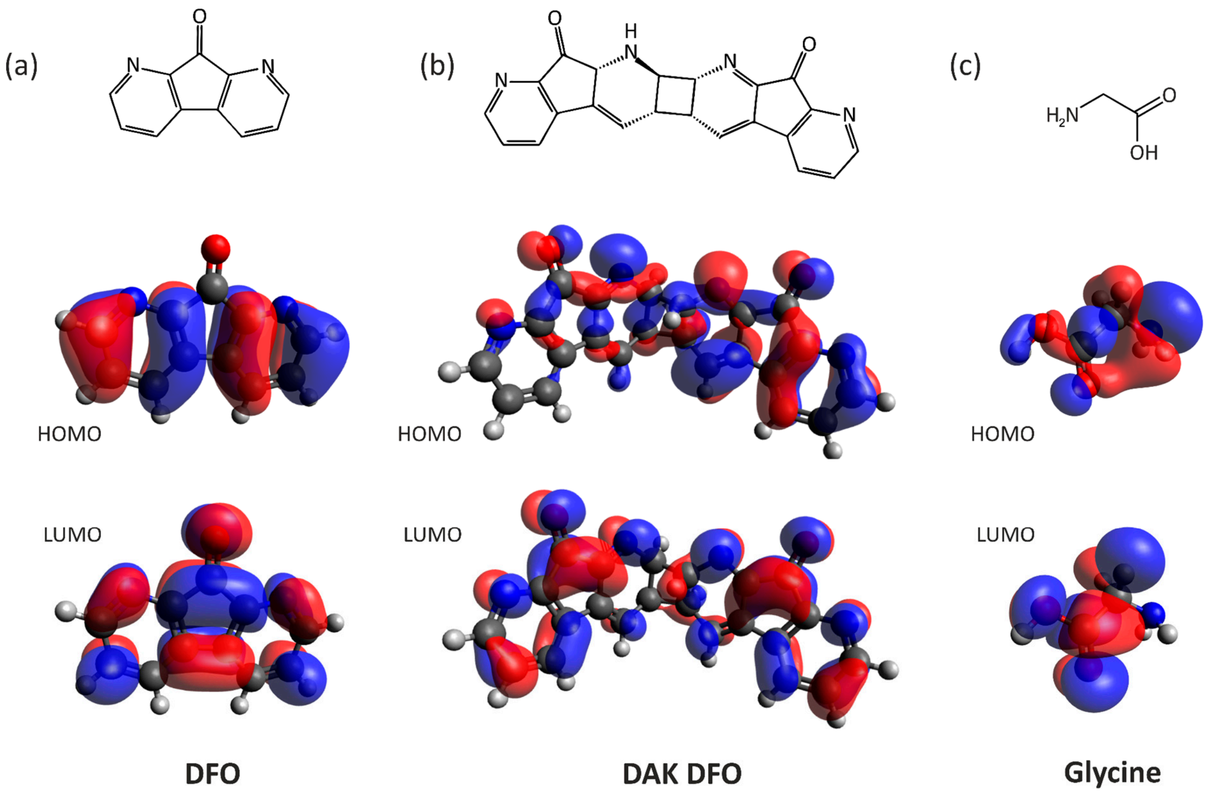

Figure S1. Chemical structures and the visualization of their HOMO-LUMO orbitals of DFO (a), DAK DFO (b), and glycine (c).

Video 1 - Reaction path for DAK DFO
